# Endogenous Proteases in Sea Cucumber (*Apostichopus japonicas*): Deterioration and Prevention during Handling, Processing, and Preservation

**DOI:** 10.3390/foods13132153

**Published:** 2024-07-08

**Authors:** Xinru Fan, Ke Wu, Xiuhui Tian, Soottawat Benjakul, Ying Li, Xue Sang, Qiancheng Zhao, Jian Zhang

**Affiliations:** 1 College of Food Science and Engineering, Dalian Ocean University, Dalian 116023, China; 2 Yantai Key Laboratory of Quality and Safety Control and Deep Processing of Marine Food, Shandong Marine Resource and Environment Research Institute, Yantai 264006, China; 3 Dalian Key Laboratory of Marine Bioactive Substances Development and High Value Utilization, Dalian 116023, China; 4 Liaoning Provincial Marine Healthy Food Engineering Research Centre, Dalian 116023, China; 5 International Center of Excellence in Seafood Science and Innovation, Faculty of Agro-Industry, Prince of Songkla University, Hat Yai 90110, Songkhla, Thailand

**Keywords:** sea cucumber, autolysis mechanism, protease inhibitors, quality deterioration, microstructure

## Abstract

The sea cucumber is an essential nutrient source and a significant economic marine resource associated with successful aquaculture. However, sea cucumbers are highly susceptible to autolysis induced by endogenous protease after postmortem, and the phenomenon of body wall “melting” occurs, which seriously affects the food quality of products and the degree of acceptance by consumers. To satisfy the growing demand for fresh or processed sea cucumbers, we must clarify the autolysis mechanism of sea cucumbers and the methods to achieve autolysis regulation. In this paper, the factors leading to the quality deterioration and texture softening of sea cucumbers are reviewed, with emphasis on enzymatic characteristics, the autolysis mechanism, the effects of autolysis on the physicochemical properties of the body wall of the sea cucumber, and the development of potential natural protease inhibitors. We aim to provide some reference in future preservation and processing processes for sea cucumbers, promote new processing and preservation technologies, and advance the sea cucumber industry’s development.

## 1. Introduction

With the increasing global demand for fresh and processed aquatic products, food and products with prime quality and safety are required for competitiveness and consumer acceptability. Deterioration, particularly in terms of quality attributes such as texture and flavor, has a detrimental effect on the palatability and market demand for processed aquatic products. Processors are focusing on quality control from the farm to the table. Consequently, storage stability, transportation, and logistics have become crucial issues.

Sea cucumber, especially Japanese sea cucumber (*Apostichopus japonicas*), a marine echinoderm species with abundant nutritional ingredients, has been recognized as a low-cholesterol, low-fat, and low-carbohydrate marine animal [1]. Sea cucumber also comprises a variety of amino acids and vitamins [2,3]. The bioactive compounds found in sea cucumber, including polysaccharides, saponins, and cerebrosides, have demonstrated multiple bioactivities, such as antioxidant effects, anti-hyperlipidemic properties, potential anti-tumor activity, and anticoagulant properties. Additionally, it may contribute to the prevention of a fatty liver [4,5,6]. Currently, there are approximately 58 species of sea cucumbers recognized as suitable for consumption [7], with the Japanese sea cucumber *(Apostichopus japonicas*) being a significant economic aquaculture and fishing species in China, Japan, and various other Asian countries and regions [8]. According to FAO [9], the global quantity of cultured Japanese sea cucumbers (*Apostichopus japonicus*) reached approximately 256,000 tons in 2022, representing a significant portion (10.1%) of other types of aquatic animal production. In China, the amount of cultivated sea cucumbers reached 248,508 tons, an increase of 11.59% compared to that reported in the previous year [10]. The growing demand for sea cucumber has increased competition within the aquaculture and food manufacturing industries. Consumer preferences for fresh and alive sea cucumbers have been expanding. 

After postmortem, fresh sea cucumber is prone to self-dissolution, known as “autolysis”, which significantly affects its storage, transportation, and preservation methods. To counter this undesirable phenomenon, blanching is typically implemented to inactivate enzymes, especially proteases, to preserve their structural integrity [8]. Sea cucumber has been subjected to several processing techniques. Over 80% of processed sea cucumber products are dried, frozen, pre-boiled, salted, and ready-to-eat [11]. Nevertheless, the continued popularity of traditional Chinese deep-processed cuisine has effectively driven innovation to maintain the freshness of raw sea cucumbers for processing sectors. 

Autolysis induced by endogenous proteases plays a crucial role in lowering the edible quality, involving texture, mouthfeel and flavor properties of post-harvested sea cucumbers. These endogenous proteases are mainly released from sea cucumbers’ body walls or intestines via the disruption of collagen fibers or the interfibrillar proteoglycan bridge in dermis tissues [12]. Subsequently, the liberated proteases can induce texture softening, epidermis damage, and tissue melting, resulting in white spots formed at the dermis. These white spots significantly impact the processing and quality of sea cucumber [13]. Autolysis typically occurs during fishing, transportation, storage and other processing steps due to several factors, such as ultraviolet irradiation (UV), cutting, mechanical damage, and other environmental parameters, such as high temperatures [12,14]. Cathepsin B, D, E, and rMMP-2 have been documented to primarily catalyze the autolysis of sea cucumber through the cleavage of collagen fibers [15,16]. Moreover, an increasing number of novel endogenous enzymes in sea cucumbers have been unearthed, and various endogenous enzymes have been found to exhibit synergistic effects on the hydrolysis of sea cucumbers [17]. Consequently, further exploration is warranted to elucidate the classification, origin, enzymatic properties, and other fundamental information pertaining to internal sea cucumber enzymes. Other unresolved causes need to be further investigated. For instance, the systematic evaluations of the proteases involved in sea cucumber autolysis are limited. Furthermore, it is not clear that multiple endogenous proteases in a sea cucumber’s autolysis and the mode of action of those proteases have not been elucidated. Therefore, further research on the sea cucumber autolytic process is still necessary.

This review mainly discusses the origins of endogenous proteases and autolysis on the ultrastructure, textural properties, and chemical composition of the sea cucumber’s body wall. The classification, characteristics, possible mechanisms of enzymatic hydrolysis mediated by endogenous proteases, and the protease inhibitors derived from natural resources were explored during sea cucumber processing and preservation. Furthermore, some future research gaps are proposed. 

## 2. Autolysis Phenomenon in Sea Cucumber

### 2.1. Autolysis in Sea Cucumber during Processing and Storage

Generally, the autolysis of sea cucumbers can be activated by external inducing factors within a short period. The culture environment (including the pH, water temperature, salt concentration, and nutritional condition), exposure to UV light, and mechanical damage (such as injury and cutting) have been known to accelerate autolysis [12,18]. The hydrolysis of structural proteins by aquatic endogenous proteases and UV-induced hydrolysis contribute to the autolysis of sea cucumbers [19,20]. One of the most significant manifestations of autolysis is “melting” [21]. There are two critical stages in the autolysis reactions of sea cucumbers during commercial production. One occurs after postharvest when raw sea cucumbers are subjected to prolonged transportation, harsh manual handling, or undesirable storage conditions. These can lead to the enhanced hydrolysis of structural proteins induced by endogenous proteases [22]. The other arises from inappropriate heating conditions. Sea cucumbers are preboiled (60 °C for 3–5 min, or 90–100 °C for 10–15 min) immediately after being caught to suddenly inactivate endogenous proteases and immobilize their shape [8]. Nonetheless, inadequate processing temperatures could retain some proteases, which still promote hydrolysis, damaging collagen fibers, causing collagen gelation, and destroying the texture [23]. However, based on the temperature-dependent autolysis (Table 1), the tenderization of the texture can be performed, and quality control has been monitored during the thermal treatment of the sea cucumber body wall (SCBW), which has been applied to achieve product specifications in the sea cucumber processing industry [24].

### 2.2. Role of UV Light in the Autolysis of Sea Cucumber

The process of apoptosis, also known as programmed cell death, is genetically regulated and involves receptor recognition and signal transduction [24]. It plays a significant role in the autolytic process of sea cucumbers. During the early stage of UVA-induced (400–320 nm) autolysis in sea cucumbers, physiological damage occurs in their body wall, leading to the accumulation of reactive oxygen species (ROS) generated through cellular respiration [24]. This triggers an oxidative stress response within the extracellular matrix, which activates the mitogen-activated protein kinase (MAPK) signaling pathway and accelerates the phosphorylation of extra-cellular regulated protein kinase (ERK), c-Jun N-terminal kinase (JNK), and p38 MAPK [20]. Furthermore, calcium imbalance occurs along with the abnormal morphology of the endoplasmic reticulum. Changes are observed in the mitochondrial membrane potential, resulting in cytochrome c release and the subsequent activation of caspase-3 and caspase-9 enzymes, which lead to DNA degradation and cell death [18,31]. A subsequent study confirmed that the injection of cytosolic calcium chelator, namely 1,2-bis (2-aminophenoxy) ethane-N,N,N′,N′-tetraacetic acid acetoxymethyl ester (pan- caspase inhibitor), into the body cavity effectively regulates the Ca^2+^ release to control apoptosis in coelomic cells [22].

Autophagy can be induced during the apoptosis process of sea cucumber cells. Essential proteins expressed by LC3-II, Atg5, PI3K, AKT, and mTOR autophagosomes cease their expression within 2 h after UV stimulation. Additionally, apoptosis-related signaling pathways such as PI3K/AKT/mTOR are also involved in sea cucumber autophagy [32]. Sea cucumber autolysis typically occurs after 6 h of UV treatment. Therefore, autophagy does not participate in the entire process of sea cucumber autolysis. Moreover, an injection of autophagy inducers can effectively delay UV-induced sea cucumber autolysis, demonstrating that autophagy plays a positive role in maintaining the body wall homeostasis of sea cucumbers [32]. However, current research on regulating sea cucumber autolysis through both apoptosis and autophagy is still experimental and has yet to be implemented in actual practice. However, further pertinent studies are warranted to effectively minimize UV-induced autolysis effectively.

### 2.3. Physicochemical Changes in the SCBW Induced by Autolysis

#### 2.3.1. Microscopic Molecular Structure

The SCBW primarily comprises collagen, glycoprotein, proteoglycan, and other soluble/insoluble compounds in the reticular formation (considered as ‘mutable collagenous tissue or MCT’). The components above constitute the essential edible part of a sea cucumber [13]. Sea cucumber collagen is classified as type I collagen, representing approximately 70% of the SCBW protein content [15]. Collagen fibrils form collagen molecules through covalent helical self-assembly and non-covalent binding via internal electrostatic interactions connected via glycosaminoglycan (GAG) [33]. The collagen fiber structure consists of a collagen fibril network with transverse connections and a proteoglycan bridging structure that collectively constitutes the sea cucumber MCT and determines its mechanical properties [34].

**Figure 1:**
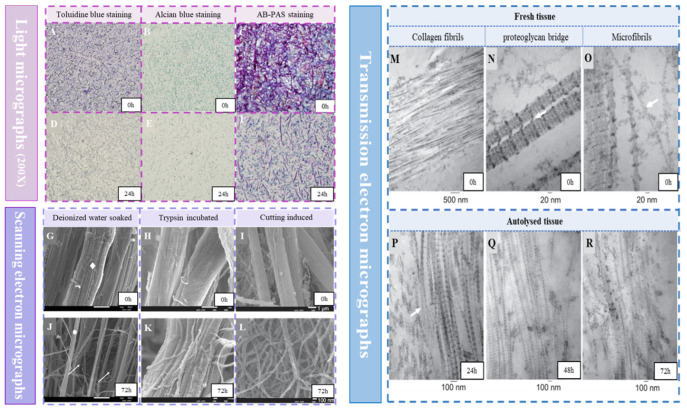
Histological analysis by light microscopy (**A**–**F**), scanning electron microscopy (**G**–**L**), and transmission electron microscopy (**M**–**R**) of fresh and autolyzed sea cucumber (*Apostichopus japonicas*) body walls (SCBWs) at room temperature (20–25 °C) for 72 h [12,37,38]. Note: AB-PAS: alcian blue-periodic acid schiff. White arrow: the location where collagen fibers fracture.

Generally, fresh sea cucumbers can undergo complete autolysis within 72 h. Ultrastructural changes in the body wall of the sea cucumber can be observed through the apparent visualization, tissue staining, and electron microscopy (Figure 1). Following the action of endogenous protease, the stratum corneum on the SCBW exfoliates and disappears, leading to the disorganization of the cell arrangement and fibrous network structure [35]. The aggrecan bridging structure between collagen fibrils was compromised, causing depolymerization into bundles or single fibrils and increasing the space between them [36]. Subsequently, the organization of collagen fibrils is loosened, while the periodic striae becomes blurred or even disappeared [13]. This process was accompanied by the degradation and dissolution of certain insoluble components in the extracellular matrix. This coincides with an increased content of water-soluble protein, soluble collagen, and glycosaminoglycan [12].

#### 2.3.2. Mechanical Properties

The mechanical properties of SCBWs, especially in textural profiles, were estimated during autolysis or the postmortem period (Table 1). Hardness and chewiness were identified as the most prominent indicators of changes in the mechanical properties during the postharvest storage of sea cucumber. The hardness was approximately half of the initial value after the first day of iced storage. By the second day, the softening process was almost complete, in which the hardness value was reduced to only one-fifth of its initial value. With increased storage time, complete autolysis typically occurs by the third day, leading to the loss of the mastication feature and a significant reduction in apparent viscosity due to complete protein hydrolysis in the SCBW [29]. Rheological evaluation has been applied to assess the mechanical properties of ready-to-eat sea cucumber and thermally treated sea cucumber. The apparent viscosity, storage modulus and loss modulus were utilized as assessment parameters. The three indices above were reduced with a prolonged refrigerated storage time, particularly the ready-to-eat sea cucumber injected with inhibitors. It exhibited varying degrees of adverse deterioration during storage [38]. 

The degree of cross-linking between the body wall fibers was determined by the hardness of the SCBW, which is generally believed to be regulated by the release of endogenous matrix metalloproteinase (MMP) inhibitor [21]. The cross-linking and depolymerization of disulfide bonds and hydrophobic interactions govern the rheological properties. The change in mechanical properties of MCT is due to alterations in the interaction between fibrils (interfibrous matrix), rather than changes in the mechanical properties of the fibrils themselves [38,39]

#### 2.3.3. Chemical Compositions

The autolysis of SCBW can be monitored by the degree of soluble fractions and oxidative indicators [13]. Due to the disintegration of the original microstructure of SCBW collagen fibers during autolysis, the GAG components were precipitated and the content of soluble collagen, such as TCA-soluble peptides, water/acid-soluble collagen, hydroxyproline, and protein fragments increased obviously [40,41]. Sea cucumber typically possesses a stable triple helical collagen structure resistant to extraction or degradation [13]. Endogenous proteases can hydrolyze collagen. The release of calcium ions activated MMP activity in response to external factors, releasing free water. Released water further promoted the occurrence of biochemical reactions [21]. However, the cysteine-containing hydrolyzed non-collagenous proteins, mainly yolk protein and actin, significantly contributed to SCBW deterioration [17,24,42].

Currently, there are two crucial processing methods for manufacturing sea cucumber products. One is the use of high-temperature and high-pressure boiling or steaming (over 100 °C, 0.15–0.21 MPa) to achieve the ripening of the SCBW followed by a water-swollen ripening process for ready-to-eat sea cucumbers [8,43]. The other is to tenderize the SCBW rich in endogenous enzymes at low temperatures and using long-term boiling technology [17]. Each type of thermal treatment usually accompanies an oxidation reaction in the SCBW [23]. Although the autolysis of fresh sea cucumber or pre-boiled frozen cucumber can affect the quality of subsequent processing, it is generally believed that intense processing leads to severe protein oxidation [24]. In particular, the process of free radical oxidation mediated by ROS has been of concern [44]. Hydroxyl radicals were generated in the SCBW heated at 37 °C for 3 h, in which protein oxidation occurred during the mild heating process [45].

## 3. The Characteristics and Mechanism of Endogenous Enzymes in the Sea Cucumber

### 3.1. Endogenous Proteases: Characteristics and Mode of Action

**Figure 2:**
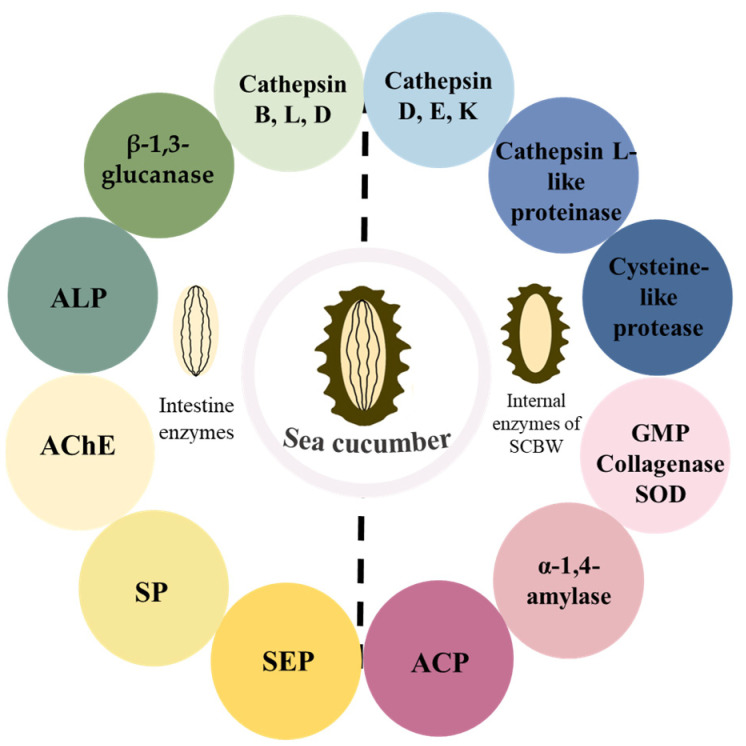
Endogenous enzymes in the intestine and body wall causing the autolysis of the sea cucumber. Note: SCBW: sea cucumber body wall, ALP: acid phosphatase, AChE: acetylcholine esterase, SP: serine protease SEP: serine endopeptidases, GMP: gelatinolytic metalloproteinase, ACP: acid phosphatase, SOD: superoxide dismutase.

Endogenous proteases causing autolysis in the sea cucumber have been isolated and purified from various tissues of sea cucumber carcasses (Figure 2). Other enzymes have also been isolated and characterized. Recently, cathepsin B [46], cathepsin D [47], β-1,3-glucanase [48], alkaline phosphatase (ALP) [30,49], acetylcholine esterase (AChE) [25], serine protease (SP) [15], serine endopeptidases (SEP) [50], and superoxide dismutase (SOD) [51] have been identified in the intestinal tract of sea cucumbers (Table 2). In addition, cathepsin L-like proteinase [27,28], cathepsin D [52], cathepsin E [52], cathepsin K [53], acid phosphatase (ACP) [54], gelatinolytic metalloproteinase (GMP) [55], cysteine-like protease [56], collagenase [57], and α-1,4-amylase [58] have been identified in the SCBW. Furthermore, a novel superoxide dismutase known as a specific deep-sea sea cucumber (*Psychropotes verruciaudatus*) copper-zinc superoxide dismutase (PVCuZnSOD) has been discovered, which has broad application potentials in the fields of bioengineering, cosmetics, and medicine [59].

These enzymes have been typically isolated and purified using anion exchange chromatography, gel filtration chromatography, the specific fluorescent substrate method, the ammonium sulfate precipitation method, and other techniques [49]. It has been found that sea cucumber primarily contains four types of proteases. Type 1 includes cysteine proteases (CP) like cathepsins B and L, which play a role in cellular protein transformation processes [61]. Moreover, cathepsin B can enhance apoptosis when the sea cucumber is under pathogenic attack [62]. The optimal activity for cathepsin B was found at pH 5.5 and 45 °C. Cathepsin L exhibits its highest activity at pH 5.0–5.5 and 50 °C. Both of these two enzymes can be inhibited by trans-epoxysuccinyl-L-leucyl-amido (4-guanidino) butane (E-64), iodoacetic acid (IAA), antipain, Cu^2+^, and Zn^2+^ [46,52]. In a fresh SCBW, cathepsin L is in cellular vacuoles released from intracellular stores and diffuses into tissues after UV stimulation for involvement in sea cucumber autolysis [35]. Type 2 consists of serine proteinase (SP). SP is separated from the intestinal tract of the sea cucumber, which effectively hydrolyzes gelatin at pH 6.0–9.0 and 30–40 °C, and is inhibited by leupeptin, Cu^2+^, Zn^2+^, Mg^2+^, Mn^2+^, Ca^2+^, and Fe^2+^ [50]. An enzyme extracted from the sea cucumber intestine, regarded as SEP, showed the maximal enzymatic activity at pH 9.0 and 40 °C [51]. Type 3 is mainly aspartic protease. Cathepsin D, the most abundant aspartic protease in lysosomes, is widely expressed in sea cucumber tissues and has been implicated in sea cucumber autolysis. It plays a crucial role in protein degradation, apoptosis, and autophagy [63,64]. Type 4 includes matrix metalloproteinases (MMPs). MMPs are the primary endogenous proteases facilitating sea cucumber autolysis by catalyzing the hydrolysis of collagen, gelatin and membrane proteins [65]. The MMPs identified in sea cucumber autolysis include tensilin, GMP, MMP, and recombinant matrix metalloproteinase-2 (rMMP-2) [55,66]. The enzyme activity can be almost completely inhibited under the combined action of enzyme inhibitors, especially EDTA and 1,10-phenanthroline [60].

### 3.2. ROS Mediated Oxidative Stress: Occurrence and Mode of Action

**Figure 3:**
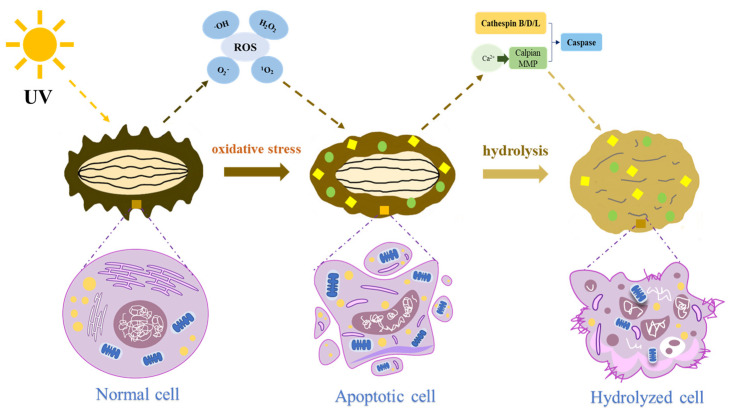
The mechanism of apoptosis participating in the autolysis process of a sea cucumber under UV induction. Note: ROS: reactive oxygen species, MMP: matrix metalloproteinase, “
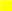
”: glycosaminoglycan (GAG), “
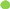
”: soluble collagen.

When sea cucumbers experience an imbalance in ROS homeostasis, a reduced ROS scavenging ability and subsequent enrichment of ROS occur, resulting in oxidative stress (OS) reactions [24]. The stress can activate endogenous proteases via two main mechanisms (Figure 3) [9]. First, through direct effects, when ROS attack, lysosomal membrane destruction takes place. As a result, permeability increases, causing the release of small molecular weight cathepsin B, cathepsin L, and cathepsin D (approximately 43 kDa) directly into the body wall of sea cucumbers, in which hydrolysis can occur. Secondly, by controlling the release of metal ions, highly oxidizing hydroxyl radicals from ROS disrupt Ca^2+^ regulation by folding proteins on the endoplasmic reticulum. This leads to an excessive cytoplasmic Ca^2+^ release, activating endogenous proteases, such as calpain and MMP [21]. Consequently, cytoskeletal protein hydrolysis occurs, resulting in cell death, while caspase activity is further accelerated, ultimately inducing apoptosis [24]. Moreover, via the regulation of cysteine release, ROS can activate MMP activities, thus achieving a bidirectional control of apoptosis [21].

### 3.3. Degradation of the SCBW

There is a complex enzyme system in sea cucumbers. Synergistic or competitive effects of various endogenous enzymes contribute to the deterioration of sea cucumber processing and storage stability [13]. Cysteine protease is involved in the hydrolysis of collagen and non-collagen proteins in the SCBW [19,42]. By disrupting the proteoglycan bridge between fibers, cysteine protease causes the partial depolymerization of collagen fibers in the sea cucumber body wall into collagen fibrils, which release glycosaminoglycan, hydroxyproline, and collagen fragments, and increase the degree of structural disorder of collagen fibers [19]. Cathepsin B, L, and K are lysosomal cysteine proteases. When the UV-induced autolysis of sea cucumber body wall occurs, the gene expression of cathepsin L is up-regulated, and the enzyme activities of cathepsin B and cathepsin L increase [14,60]. Cathepsin B has both endopeptidase and exopeptidase activities capable of degrading collagen, connective tissue proteins, and certain natural enzymes [67]. The content of cathepsin L is higher in the sea cucumber epidermis than in the dermis. Its density and distribution are proportional to the sea cucumber autolysis rate and extent [27,35]. Under UV induction, cathepsin L is released from the cell and makes contact with the extracellular substrate collagen fiber, which decomposes into collagen fibrils, thus weakening the sea cucumber texture [53]. Cathepsin K can induce multi-site degradation on stable collagen triple helix structures while degrading the body’s elastic fiber [68,69]. In the autolysis process of sea cucumber via the dissolution of lysosomes and the release of cathepsin, cathepsin K can hydrolyze the collagen of the SCBW under acidic conditions and the non-collagen part under neutral conditions [53]. 

Serine protease (SP) is a kind of protease with a serine group in active sites, which can cleave protein chains and activate MMPs [15]. Nevertheless, SP can inactivate MMPs as tissue inhibitors [15]. SP extracted from the intestine of sea cucumber will destroy serine residual sites in crude collagen fibers, reduce the cross-linking of sea cucumber collagen fibers, and soften the SCBW [15]. Trypsin is an SP, which can decompose the proteoglycan bridge structure between collagen fibrils during the autolysis process of sea cucumber, resulting in the release of water-soluble glycosaminoglycan and protein fragments of sea cucumber [37].

Cathepsin D and cathepsin E are found in sea cucumbers and belong to the aspartic protease family [70]. Cystatin D has been identified in lysosomes and is widely distributed in various tissues and cells of mammals, especially in sea cucumbers, with the highest content in the intestine followed by the muscle and body wall [71]. Cathepsin E is a non-secretory intracellular protease with a relatively limited distribution. It exists only in some sea cucumber tissues [52]. Cystatin D participates in protein degradation under the strong acid conditions of lysosomes with strong fibrin and fibrinogen degradation activity and the hydrolysis of the carboxyl COOH end peptide of collagen [63]. Cathepsin D and E have serine and cysteine residues that regulate their activity, which may be involved in protein degradation in the autolysis process of the sea cucumber [52]. In addition, the relative expression level of the cathepsin D gene is significantly up-regulated during the autolysis of sea cucumber, and the purified recombinant sea cucumber cathepsin D has been confirmed to promote the degradation of sea cucumber muscle [64].

MMP primarily functions to degrade various proteins, such as collagen, mucin, glycoprotein and other components of the extracellular matrix [72]. Tensilin was isolated, purified, and identified as a member of the MMP family from the body wall of the North Atlantic sea cucumber (*Cucumaria frondosa*) [66]. Tensilin is a collagen fiber-binding protein that interacts with stiparin, inhibiting its ability to bond collagen fibers [73]. Gelatinase has also been isolated and identified from the SCBW. Its inhibition can effectively terminate the dissolution of soluble proteins in the autolysis process of sea cucumber [55]. The proteoglycan bridge can be destroyed by MMPs, resulting in the complete depolymerization of collagen fibers into smaller collagen fibril bundles and fibrils. Additionally, MMPs cause partial degradation of collagen fibers by releasing soluble hydroxyproline and pyridine crosslinking products [26]. Among the members of the MMP family, rMMP-2 is a distinctive protease that efficiently catalyzes the hydrolysis of type I collagen and gelatin, leading to the decomposition of collagen fibers into fibrils in the SCBW [41].

## 4. Inhibitors for Sea Cucumber Endogenous Enzyme Proteases

Endogenous proteases, especially MMP, are pivotal enzymes in sea cucumber autolysis, particularly at the body wall, causing the quality loss during processing, transportation, and short-term storage [41]. Therefore, the inhibition of SCBW autolysis is mainly achieved through chelators of metal ions, which are required for MMP activity. Moreover, some natural extracts and organic acids are considered to be sea cucumber endogenous protease inhibitors. EDTA and 1,10-phenanthroline, as bivalent metal chelating agents, have demonstrated a positive inhibitory effect toward SCBW hydrolysis [57]. Under the stimulation of external conditions, the content of Ca^2+^ in the dermis of sea cucumber increases, leading to the synchronous activation of the MMP function. Adding an MMP inhibitor (1,10-phenanthroline) allows the chelation of Ca^2+^, thus inhibiting MMP activation, and delaying the autolysis of the sea cucumber [21]. Previous studies have revealed that UV irradiation induces the release of Ca^2+^ from the endoplasmic reticulum and activates the apoptosis pathway in sea cucumbers [20]. This results in the upregulation of casase-3 and casase-9 associated with apoptosis-related metabolic enzymes, ultimately leading to coelom cell apoptosis [17]. However, injecting a cytoplasmic calcium chelator into a sea cucumber effectively maintains cellular Ca^2+^ homeostasis and inhibits sea cucumber autolysis until 48 h have passed by controlling the cell apoptosis processes [22]. 

Tea polyphenol has the chelating ability of metal ions [74]. Oxalic acid is an organic acid with strong metal-chelating characteristics [75]. When fresh sea cucumber was soaked in 0.2% (*w*/*v*) tea polyphenol and 0.2% (*w*/*v*) oxalic acid solution, the endogenous enzyme activity could be considerably inhibited by chelating calcium ions, which could dramatically impede the degradation and oxidation of sea cucumber proteins during refrigeration storage, thus reducing the release of soluble proteins, glycosaminoglycans and hydroxyproline in sea cucumber. Consequently, the textural deterioration (softening) of sea cucumber quality can be delayed 48 h (total shelf life of 72 h) [38]. Furthermore, there is growing interest in brown algae feedstocks rich in polyphenols sourced from marine resources as potential inhibitors. Extracts of phlorotannins obtained from brown algae (*Ascophyllum nodosum*) have been demonstrated to promote the cross-linking and aggregation of endogenous proteases within sea cucumbers while reducing the thermal stability of proteases. Those extracts could inhibit enzymes in a dose-dependent manner [76]. During subsequent heating processes (37 °C for 3 h), these polyphenol and phlorotannin extracts can effectively hinder oxidation generation by eliminating free radicals, mitigate protein side chain modification, enhance thermal stability properties of the body wall texture, and delay protein degradation during processing [77,78]. The polyphenol oxidase-mediated (-)-epigallocatechin gallate has been verified to inhibit CP activities by modifying the enzyme structure and promoting protein oxidation [79]. The coelomic fluid isolate from sea cucumber has also been demonstrated to enhance the inhibition rate of SP and CP in sea cucumber up to 56.2–87.2%, which can serve as a potential source for the preparation of inhibitors [80].

## 5. Conclusions

Sea cucumbers are susceptible to self-degradation and tissue damage, ultimately causing evisceration and significant economic losses. Endogenous enzymes, particularly proteases, in sea cucumbers are primarily involved in autolysis. Additionally, UV-induced stress also triggers the autolysis of sea cucumbers. To tackle these problems, the specific inhibitors, which can maintain cellular homeostasis and inhibit proteolysis, can be employed to retard the deterioration of sea cucumber quality. Various natural products and chemical compounds, such as protease inhibitors, can suppress the activity of autolytic enzymes in sea cucumbers. Future research should focus on developing effective protease inhibitors to stabilize sea cucumber quality during storage and processing. Optimizing inhibitor application methods, understanding the underlying biochemical mechanisms, and integrating sustainable processing technologies are crucial steps toward enhancing sea cucumber production’s economic viability and competitiveness. These efforts will advance scientific knowledge and support the seafood industry in meeting growing market demands sustainably.

## Figures and Tables

**Table 1:** A summary of the key endogenous enzymes, related inhibitors, and their effects on physicochemical properties of sea cucumber (*Apostichopus japonicas*) during processing and preservation.

Enzymes Species	Endogenous Enzymes	Heating/Storage Condition	Target Protein	Inhibitors	Metal ions (Activation (↑)/Inactivation (↓))	Molecule Weight	Textural Properties Decrease (↓)	References
Metalloprotease (MMP)	Matrix metalloproteinase-2 (MMP-2)	25 °C for 72 h	Type I collagen	NaN_3_ (0.03%), PMSF (5 mM), E-64 (0.1 mM) and CaCl_2_ (5 mM)	-	-	-	[16]
	Gelatinolytic metalloproteinase (GMP)	37 °C for 18 h	Collagen bands (α1 and β)	EDTA (10 mM); EGTA (10 mM); 1,10-phenanthroline (10 mM)	Ca^2+^ (↑)	45 kDa	-	[25]
	Collagenase type I	30 °C for 72 h	Collagen fibers	-	-	-	-	[13]
	Matrix metalloproteinase (MMP)	Boiled (10 min) sea cucumber stored at 4 °C for 60 d	Extracellular matrix in the dermis; Interfibrillar proteoglycan bridges	EDTA Na_2_ (10 mM); 1,10-phenanthroline (10 mM)	Ca^2+^ (↑)	-	Shear force (↓); hardness (↓); elasticity (↓); cohesiveness (↓); chewiness (↓); coverability (↓)	[21,26]
Cysteine protease (CP)	Cysteine proteinases	37 °C for 2~72 h	Collagen fibers	PMSF (1 mM), 1,10-phenanthroline (1 mM), and E-64 (0.5 mM)	-	-	-	[19]
	Cathepsin L-like proteinase	20~70 °C for 30 min	The epidermis of the body wall	E-64 (0.1 mM); Indoacetic acid (1 mM); Antipain (1 mM)	Zn^2+^ (↓); Cu^2+^ (↓); Fe^2+^ (↓)	30.9 kDa	-	[27,28]
	Cathepsin L and Cathepsin B	4 °C for 8 days	Body wall	-	-	-	Hardness (↓); chewiness (↓); springiness (↓); adhesiveness (↓)	[29]
Serine proteinase (SP)	High alkaline protease (serine protease-like)	4~65 °C for 1 h	-	EDTA (10 mM); PMSF (5 mM)	Ca^2+^ (↑); Mg^2+^ (↑); Cu^2+^ (↑); Zn^2+^ (↓); Hg^2+^ (↓);	20.6 kDa; 39.1 kDa; 114.1 kDa;	-	[30]
	Serine proteinase	37 °C for 12 h	Collagen bands (α, β and γ)	Pefabloc SC (2 mM); Benzamidine (5 mM)	-	34 kDa	-	[15]
Mixed protease system	Cysteine (cathepsins, calpains, caspases and proteasomes), serine protease and MMP	37 °C for 1~36 h	Body wall	E-64 (0.1 mM); PMSF (1 mM); 1,10-phenanthroline (1 mM)	-	-	Hardness (↓); chewiness (↓)	[17,24]

**Table 2:** Characteristics of various endogenous enzymes in sea cucumber.

Sea Cucumber Species	Enzyme Types	Extracted Position	Molecule Weight (kDa)	Optimum Temperature (°C)	Optimum pH	Activator	Inhibitors	References
Janpanese sea cucumber (*Apostichopus japonicus*)	Cathepsin B	Intestine	49	45	5.5	DTT, L-Cys, EGTA, EDTA	E-64, IAA, Antipain, Cu^2+^, Ni^2+^, Zn^2+^	[46]
	Cathepsin L	Intestine	-	48	4.4	-	E-64, IAA, Antipain	[60]
	Cathepsin D	Intestine	-	50	3.0	Cd^2+^	E-64, IAA, Pepstatin A, Fe^3+^, Fe^2+^	[47]
		Body wall		60	5.0	DTT	Pepstatin A, PMSF, Zn^2+^,Cu^2+^, Fe^2+^,Fe^3+^, Mn^2+^	[51]
	β-1,3-glucanase	Intestine	37.5	40	5.5	Mn^2+^	Cu^2+^, Ag^+^, Zn^2+^, Fe^2+^, Ca^2+^	[48]
	ACP	Body wall	148	40	4.0	Mg^2+^	Zn^2+^, Cu^2+^, Fe^2+^, Fe^3+^	[54]
	ALP	Intestine	166 ± 9	40	11.0	Mg^2+^	Zn^2+^, Ca^2+^, EDAT	[49]
	AChE	Intestine	68	35	7.5	-	Eserine, BW284C51	[25]
	SP	Intestine	34	35-40	6.0–9.0	EDTA	Leupeptin, Cu^2+^, Zn^2+^, Mg^2+^, Mn^2+^, Ca^2+^, Fe^2+^	[15]
	SEP	Intestine	-	40	9.0	-	PMSF	[50]
	Cathepsin L-like proteinase	Body wall	30.9	50	5.0–5.5	DTT, L-Cys, EDTA	E-64, IAA, Antipain, Zn^2+^, Fe^2+^, Cu^2+^	[27]
	Cathepsin E	Body wall	-	40	4.0	DTT	Pepstatin A, PMSF, Fe^3+^, Fe^2+^, Cu^2+^	[52]
	Cathepsin K	Body wall	-	50	5.0	Mg^2+^, DTT, L-Cys,	E-64, IAA, Antipain, PMSF, EDTA, Zn^2+^, Fe^2+^, Fe^3+^, Cu^2+^	[53]
	Cysteine-like protease	Body wall	35.5	50	7.0	L-cysteine hydrochloride	Antipain, leupeptin, Cu^2+^, Mg^2+^, Fe^2+^, Fe^3+^	[56]
	GMP	Body wall	45	40-45	8.0–9.0	Ca^2+^, Ba^2+^	EDTA, EGTA, 1,10- phenanthroline	[55]
	α-1,4-amylase	Body wall	420	80	9.0	Cu^2+^, Mg^2+^	Mn^2+^, K^+^, Fe^3+^	[58]
	Collagenase	Body wall	45	40	8.0	Mn^2+^	EDTA, 1,10-phenanthroline	[57]
	SOD	Body wall	-	30-60	4.0	Ca^2+^, Zn^2+^	H_2_O_2_, Fe^2+^	[51]
Deep-sea sea cucumber (*Psychoropotes verruciaudatus*)	PVCuZnSOD	-	15	20	4–11	Mg^2+^, Ni^2+^	Mn^2+^, Cu^2+^, Zn2^+^, Co^2+^	[59]

Note: ACP: acid phosphatase, ALP: acid phosphatase, AChE: acetylcholine esterase, SP: serine protease, SEP: serine endopeptidases, GMP: gelatinolytic metalloproteinase, SOD: superoxide dismutase, PVCuZnSOD: Psychropotes verruciaudatus copper-zinc superoxide dismutase, DTT: Dithiothreitol, L-Cys: L-Cysteine, EDTA: ethylenediamine tetraacetic acid, EGTA: ethylene glycol tetraacetic acid, E-64: trans-epoxysuccinyl-L-leucyl-amido (4-guanidino) butane, IAA: iodoacetic acid, PMSF: phenylmethylsulfonyl fluoride, BW284C51: 1,5-bis(4-allyldimethylammonium phenyl)-pentan-3-one dibromide.

## Data Availability

No new data were created or analyzed in this study. Data sharing is not applicable to this article.
